# The Fifth International Survey of Critical Care Nursing Organizations: Implications for Policy

**DOI:** 10.1111/jnu.12599

**Published:** 2020-10-22

**Authors:** Ged Williams, Paul Fulbrook, Ruth Kleinpell, Laura Alberto

**Affiliations:** ^1^ Chief Nursing Officer Mafraq Hospital United Arab Emirates and Adjunct Professor School of Nursing & Midwifery Griffith University Queensland Australia; ^2^ Professor of Nursing School of Nursing, Midwifery & Paramedicine Australian Catholic University, Nursing Director Nursing Research & Practice Development Centre The Prince Charles Hospital, Brisbane, Australia and Honorary Professor Faculty of Health Sciences University of the Witwatersrand Johannesburg South Africa; ^3^ Assistant Dean for Clinical Scholarship and Professor Vanderbilt University School of Nursing, TN and Professor Rush University College of Nursing Chicago USA; ^4^ Professor School of Nursing Universidad del Salvador Argentina

**Keywords:** Critical care, education, international, nursing, professional issues, survey, workforce

## Abstract

**Purpose:**

To examine the activities, concerns, and expectations of critical care nurses and professional critical care nursing organizations worldwide.

**Design:**

A descriptive survey methodology was used. This study is the fifth worldwide quadrennial review of its type to monitor variations in critical care nursing needs and provide robust evidence to inform policy related to critical care nursing practice.

**Methods:**

The fifth World Federation of Critical Care Nurses international survey of critical care nursing organizations was emailed to potential participants from countries with critical care nursing organizations or known critical care nurse leaders. Data were collected online. Responses were entered into SPSS version 23 software (IBM Corp., Armonk, NY, USA) and analyzed by geographical region and national wealth group.

**Findings:**

Eighty‐two national representative respondents participated in the survey, of whom two thirds (*n* = 56, 68%) had an established critical care nursing organization in their country. The five most important issues identified were working conditions, teamwork, staffing levels, the need for formal practice guidelines and competencies, and wages. The top five critical care nursing organization services that were considered to be of most importance were professional representation, as well as provision of workshops and education forums, national conferences, practice standards and guidelines, and local conferences. The most important contributions expected from the World Federation of Critical Care Nurses were standards for clinical practice and professional practice, international conferences, professional representation, and study and education grants.

**Conclusions:**

The results highlight priority areas for critical care nursing and reinforce the need to address factors that can inform critical care nursing policy and practice. Results of this survey should be incorporated into strategic action plans at the national and international levels.

**Clinical Relevance:**

Nursing leaders, policymakers, and other interested stakeholders should consider these findings when planning critical care workforce requirements. Interested parties should work collaboratively to inform recommendations for further policy and action.

The World Federation of Critical Care Nurses (WFCCN) was established in 2001 and became a member organization of the International Council of Nurses in 2007. Its primary function is to provide leadership, guidance, and advice to critical care nursing organizations (CCNOs) and critical care nurses (CCNs) throughout the world. In addition, the WFCCN provides an important advocacy role on behalf of critically ill patients, their relatives, and their carers, and contributes an active voice on many international forums to help inform and guide critical care practices globally.

In 1999–2001, the first worldwide survey of all known CCNO and international CCN leaders was used to inform the original charter of the WFCCN and the priority needs of the profession (Williams et al., 2001). Every 4 years, a similar survey has been conducted and results published to ensure the profession is kept up to date with the latest synthesis of opinions and priority areas as identified by CCN leaders from around the world (Williams, Fulbrook, Kleinpell, Schmollgruber, & Alberto, 2015).

## Background

Previous global surveys have identified some consistent themes that tend to dominate the concerns of CCN leaders: workforce, education and training, representation and advocacy, and communication (use of conferences, websites, journals, and newsletters). Other issues that have emerged over time include considerations surrounding the need for teamwork, research, clinical protocols, and practice standards, and concerns regarding ethical issues such as end‐of‐life care and decision‐making (Williams et al., 2015). In North America, Stelfox et al. (2015) found that critical care providers identified end‐of‐life care, early mobilization, and strategies to preserve patient sleep as the three most important priorities for improving quality and value of critical care. In Europe, Blackwood, Albarran, and Latour (2011) used a Delphi technique to survey 110 European CCN opinion leaders and identified five broad priorities: patient safety, impact of evidence‐based practice on outcomes, impact of workforce on outcomes, well‐being of patients and relatives, and impact of end‐of‐life care on staff and practice.

Healthcare system leaders, on the other hand, tend to emphasize the need for greater access to beds (Murthy, Leligdowicz, & Adhikari, 2015), reducing length of stay, and improving patient and family experiences as important priorities for critical care (NHS Wales, 2013). Recently, there has been an emphasis on the provision of “essential emergency and critical care” for all critically ill patients worldwide (Schell et al., 2018), based on principles of universal health coverage (Jamison et al., 2018).

Priorities for critical care nursing draw on many perspectives. National and regional health systems, individual hospitals, and clinician groups have been represented in studies articulating their respective views of critical care services. However, very few take a global perspective, and this is what allows the WFCCN to hold its place as an important advocate to explain issues and priorities as well as the need for change in practice at the global level. Thus, the aim of this study was to examine the activities, concerns and expectations of critical care nurse leaders and national critical care nursing organizations to provide a contemporary global perspective and compare it with trends identified in our previous studies.

This fifth worldwide survey of the activities, priorities, and needs of CCNs globally comes at a time when the world is rapidly changing. Understanding CCN leaders’ views will assist the WFCCN and other policy leaders to support the profession and drive advocacy that is responsive to the changing and diverse global needs of the profession and the community.

## Methods

An online survey was conducted using a structured questionnaire. Ethical approval for the study was provided by the WFCCN Council. The survey was considered to be low risk, as it did not involve patients and did not require the collection of individually identifiable data. Consent was implied by voluntary submission of the questionnaire. As with previous surveys conducted using this approach, for the above reasons and because the respondents were individual professionals that were not representing healthcare institutions, institutional review board approval was deemed unnecessary.

### Sample

As in previous surveys, the WFCCN accessed its extensive international network utilizing a purposive sampling method to identify a nonprobability sample of CCNOs and CCN leaders worldwide to respond to the survey. In countries where there was no known CCNO, one or more CCN leaders who could represent their national perspective were identified; at the time, the WFCCN had contacts in more than 90 countries. In countries with no existing CCN contacts, we called on the participating contacts from the same region to help us to identify reliable and knowledgeable CCN leaders in these countries (a modified snow‐balling approach). The WFCCN has five regional federation partners in Africa, Europe, Latin America, South Asia, and South‐East Asia who also assisted in identifying reliable contacts in their regions. Where English or Spanish was not the first language of identified respondents, efforts were made collaboratively between the researchers and the respondent to identify a bilingual translator to help with the response as necessary.

### Survey Tool

The questionnaire was based on those used in previous WFCCN surveys. After consultation with WFCCN members regarding the contemporary content of the questionnaire, it was revised with several new questions added around the educational and research priorities. The tool was piloted through a convenience sample of eight experienced WFCCN members. The main purpose of the pilot was to check that all questions and statements were easily understandable and there was no ambiguity. Only minor wording revisions were made following the pilot. The final English version was forward translated into Spanish by a bilingual member of the team with experience in translation procedures.

The questionnaire comprised 32 items in five sections. Section 1 collected demographic information and section 2 sought information about CCNOs and the services they provided. In section 3, respondents were asked to rate the importance of 16 services or activities commonly provided by CCNOs, using a 10‐point ordinal scale (range 1–10; 1 = *not at all important*, 10 = *very important*). In section 4, using the same 10‐point scale, respondents were asked to rate the importance of 14 critical care nursing issues (identified in previous surveys). Additionally, respondents were asked to identify strategies that had been used by their CCNO (if applicable) to respond to specific issues. In section 4, a range of questions was posed that focused mainly on aspects of educational preparation and research priorities. The final section explored respondents’ rating of the importance (10‐point scale) of 11 services and activities provided by the WFCCN. The respondents were also asked to identify other services or activities and areas of nursing practice that would benefit from position statements or guidelines that could be provided by the WFCCN.

### Data Collection and Analysis

Potential respondents in 104 countries were initially contacted by email (in several countries multiple contacts were emailed) and requested to complete the questionnaire via an online survey tool (Survey Monkey, San Mateo, CA, USA^1^). Individuals who were not familiar with Survey Monkey or challenged by language or translation were guided by the researchers via email to help them find suitable solutions to complete the task. Data were collected for a 5‐month period from April to August 2017. Response data were imported into SPSS version 23 software (IBM Corp., Armonk, NY, USA). For analysis, respondents were grouped by geographical region and relative wealth. The latter group was categorized by national gross domestic product and per capita purchasing power parity (GDP PPP) (Central Intelligence Agency, 2018). Descriptive and inferential tests were used to describe the sample and examine relationships. Significance was set at *p* < .05. A simplified thematic analysis was used to synthesize qualitative information.

## Results

From the initial contact sample, 106 responses were returned. Following removal of duplicΑΑates, responses from 82 countries were included in the analysis, giving a country response rate of 77% (82 of 104). Of these, 9 questionnaires were not completed. The largest group was from Europe (*n* = 20, 24%), with the remainder from the Americas (*n* = 18, 22%), Africa (*n* = 17, 21%), the Asia Pacific (*n* = 14, 17%), and the Middle East (*n* = 13, 16%) regions. Twenty countries (24%) were in the poorest third of countries in the world, with a GDP PPP ranging from $US900 to $US7,200 (Central Intelligence Agency, 2018). There were 31 middle‐wealth countries (37%; GDP PPP $US7,700–24,900) and 31 high‐wealth countries (39%; GDP PPP $US27,500–124,900). Among those countries represented, two thirds (*n* = 13) of African nations were in the lower third wealth group, with the remainder (*n* = 4) in the middle third. The majority of respondents from the Americas were in the middle third wealth group (*n* = 14, 78%) and there were no respondents from low‐wealth countries in Europe (top third, *n* = 16, 80%). In the Asia Pacific and Middle East regions, the majority of respondents were from countries within the top two wealth groups (*n* = 10, 71%; *n* = 11, 85%, respectively; Table S1).

### Issues for Critical Care Nurses

Respondents rated all of the critical care nursing issues as being important with the lowest mean score being 8.57 (Table 1). Working conditions was considered to be the most important issue (mean score 9.51, *SD* .84), followed by teamwork, staffing levels, formal practice guidelines, wages, and access to quality education programs. High‐wealth countries scored lower than middle‐ and low‐wealth groups on all but one item (teamwork).

**Table 1:** Critical Care Nursing Issues by Relative Wealth (*n* = 75)

Issue	Rank 2013	Rank 2017	Overall score			Wealth group mean score		
			Mean (*SD*)	95% CI	Range	Top third	Middle third	Lower third
Working conditions	1	= 1	9.51 (.84)	9.31–9.70	7–10	9.15	9.73	9.67
Teamwork	5	↑ 2	9.47 (1.11)	9.21–9.72	3–10	9.44	9.60	9.28
Staffing levels	3	= 3	9.43 (.96)	9.21–9.65	5–10	9.26	9.47	9.61
Formal practice guidelines/ competencies	2	↓ 4	9.32 (1.07)	9.07–9.57	5–10	8.93*	9.43	9.72*
Wages	6	↑ 5	9.13 (1.32)	8.83–9.44	4–10	9.04	9.17	9.22
Access to quality educational programs	4	↓ 6	9.13 (1.44)	8.80–9.47	2–10	8.96	9.03	9.56
Extended/advanced practice	7	= 7	9.05 (1.23)	8.77–9.34	3–10	8.93	8.97	9.39
Work activities/roles	8	= 8	8.99 (1.33)	8.68–9.29	4–10	8.63	9.23	9.11
Relationships with doctors	11	↑ 9	8.85 (1.44)	8.52–9.18	3–10	8.59	9.10	8.83
Use of technologies	10	= 10	8.69 (1.81)	8.28–9.11	1–10	8.44	8.70	9.06
Formal credentialing processes	12	↑ 11	8.69 (1.99)	8.24–9.15	1–10	7.41*	9.30*	9.61*
Facilities and equipment	9	↓ 12	8.63 (1.91)	8.19–9.07	1–10	8.07	8.70	9.33
Relationships with other healthcare groups	14	↑ 13	8.59 (1.66)	8.20–8.97	3–10	8.26	8.87	8.61
Relationships with other nursing organizations	13	↓ 14	8.57 (1.91)	8.13–9.01	2–10	8.33	8.60	8.89

CI = confidence interval; SD = standard deviation.

* *p* < .05.

When critical care nursing issues were compared by wealth groups using one‐way analysis of variance (ANOVA), statistically significant differences were found between two issues. Due to significant variance in the homogeneity of scores, Welch’s F test was used to examine differences in formal practice guidelines or competencies (*F* [2, 46.26] = 4.04, *p* = .024) and formal credentialing processes (*F* [2, 44.60] = 8.28, *p* = .001). Post hoc tests using the Games‐Howell statistic revealed that high‐wealth countries (mean score 8.93, *SD* 1.44) scored significantly lower than low‐wealth countries (mean score 9.72, *SD* .46; *p* = .030) but not middle‐wealth countries (mean score 9.43, *SD* .82; *p* = .252) in their rating of formal practice guidelines or competencies as an issue. Additionally, in their rating of formal credentialing processes as an issue, high‐wealth countries (mean score 7.41, *SD* 2.68) scored significantly lower than both middle‐wealth (mean score 9.30, *SD* .95; *p* = .004) and low‐wealth countries (mean score 9.61, *SD* .70; *p* = .001; see Table 1).

When CCN issues were compared by geographical region using ANOVA, statistically significant differences were found between scores for working conditions (*F* [4, 33.03] = 3.37, *p* = .020) and formal credentialing processes (*F* [4, 31.23] = 3.84, *p* = .012). Post hoc tests (Games‐Howell) revealed that the only statistically significant difference in scores for working conditions was between African and Middle East nations, with the former group rating the issue higher (mean 9.87, *SD* .52) than the latter (mean 8.92, *SD* .90; *p* = .034). Additionally, the only statistically significant difference in scores for formal credentialing processes was between African and European nations, with the former group rating the issue higher (mean 9.67, *SD* .62) than the latter (mean 7.67, *SD* 2.40; *p* = .021).

Most respondents identified and described other major services and activities that would be beneficial for CCNs in their country (Table S2) or clinical and professional issues with written position papers provided by WFCCN (Table S3). A total of 183 individual issues were named and were systematically grouped into themes. With respect to services and activities, the major themes that were identified included advocacy, collaboration, and representation; communication and networking; research support; supporting critical care education and practice; and supporting publication. With respect to position statements, major themes included advanced practice, clinical guidelines, competency, education and practice standards, regulation, research, and workforce guidelines.

### Critical Care Nursing Organizations

Two thirds of respondents (*n* = 56, 68%) reported that they had a CCNO in their country. Of these, most were members or associate members of the WFCCN (*n* = 39, 70%). Fifty‐one CCNOs provided information about their membership numbers. Thirteen CCNOs had up to 100 members, 28 had between 100 and 1,000 members, and 11 had over 1,000 members. In total, these CCNOs represented 43,728 members.

The service and activities provided by CCNO were ranked by importance (Table 2). Although provision was variable, 9 of the top 10 ranked services were provided by the majority of CCNOs. When compared by wealth group using ANOVA/Welch test, the importance of several services or activities were rated significantly differently (see Table 2). Post hoc tests revealed that middle‐wealth countries valued initiating, conducting, and leading research studies significantly more than high‐wealth countries (*p* = .034). Training or skill acquisition courses were valued significantly less by high‐wealth countries than both middle‐wealth countries (*p* = .007) and low‐wealth countries (*p* = .005). The importance of standards for educational courses was rated higher by low‐wealth countries than both middle‐wealth countries (*p* = .041) and high‐wealth countries (*p* = .004). The provision of study or education grants was considered significantly more important by low‐wealth countries than high‐wealth countries (*p* = .004).

**Table 2:** Provision and Importance of CCNO Services and Activities (*n* = 75)

Activity or service		Importance of activity/service							
Description	Provided by CCNO (*n* = 53)	Rank 2013 (*n* = 55)	Rank 2017 (*n* = 75)	Overall score (*n* = 75)			Mean score by wealth group		
				Mean (*SD*)	95% CI	Range	Top third (*n* = 27)	Middle third (*n* = 30)	Lower Third (*n* = 18)
Professional representation	47 (89%)	3	↑ 1	9.45 (1.51)	9.11–9.80	1–10	8.93	9.73	9.78
Workshops/education forums	42 (79%)	6	↑ 2	9.44 (1.18)	9.17–9.71	2–10	9.04	9.57	9.83
National conferences	46 (87%)	1	↓ 3	9.36 (1.47)	9.02–9.70	1–10	9.19	9.40	9.56
Practice standards/guidelines	30 (57%)	4	= 4	9.35 (1.80)	8.93–9.76	1–10	8.37	9.83	10.00
Local conferences	46 (87%)	5	= 5	9.17 (1.72)	8.78–9.57	1–10	8.48	9.60	9.83
Website	38 (72%)	2	↓ 6	9.15 (1.70)	8.76–9.54	1–10	8.89	9.37	9.17
Initiate, conduct, or lead research studies	32 (62%)	8	↑ 7	8.95 (1.82)	8.53–9.37	2–10	8.15*	9.47*	9.28
Training/skill acquisition course	32 (60%)	9	↑ 7	8.95 (2.27)	8.42–9.47	1–10	7.56*	9.70*	9.78*
Standards for educational courses	27 (51%)	7	↓ 9	8.89 (2.08)	8.42–9.37	1–10	7.96*	9.13*	9.89*
Research grants	21 (40%)	13	↑ 10	8.71 (2.03)	8.249.17	3–10	8.56	8.70	8.94
Study/education grants	20 (38%)	12	↑ 11	8.61 (2.02)	8.15–9.08	3–10	7.74*	8.87	9.50*
Newsletter	26 (49%)	14	↑ 12	8.51 (2.11)	8.02–8.99	1–10	7.93	9.17	8.28
Journal	26 (49%)	10	↓ 12	8.51 (1.81)	8.09–8.92	2–10	8.37	8.73	8.33
Credentialing or accreditation process	13 (25%)	11	↓ 14	8.40 (2.67)	7.83–8.97	1–10	6.48*	9.47	9.50
Travel scholarships	14 (26%)	15	= 15	7.96 (2.18)	7.46–8.46	1–10	8.11	7.50	8.50
Industrial/union representation	20 (37%)	16	= 16	6.92 (3.00)	6.24–7.60	1–10	5.89	7.37	7.72

CI = confidence interval.

* *p* < .05.

### Policy and Professional Leadership

Of the 75 CCNOs that responded, 67 (89%) identified the need for national policies or guidelines. Many of the same themes were raised regarding workforce and staffing ratios, education and certification requirements for specialist CCNs, as well as access to ongoing education and practice standards generally. The most common specific issue to be raised was the need for greater guidance on end‐of‐life decision making and care. Others included antibiotic stewardship, infection prevention practices, and consistent data collection for benchmarking quality performance (Tables S2 and S3).

### Research Priorities

Respondents suggested 322 areas of research that they considered valuable. These were grouped thematically. The most frequently cited research area (*n* = 41) was related to critical care staffing and workload and workforce. Various aspects of infection control were the second most commonly cited research area (*n* = 28), followed by issues around nursing education (*n* = 26), safety and quality (*n* = 23), and nursing roles and advanced practice (*n* = 22).

### Role of the WFCCN

Eleven services and activities provided by the WFCCN were ranked by importance (Table S4) and compared by wealth group using ANOVA/Welch test. Although there was a trend for high‐wealth countries to rate the importance of services and activities lower than middle‐ and low‐wealth countries, only individual membership of the WFCCN was statistically significantly different (*F* [2, 40.49] = 5.37, *p* = .009). Standards for clinical practice, international conferences, and standards for professional practices were ranked most important of the options provided. Other common needs included provision of assistance to CCNs in countries that did not have a CCNO and help to support their establishment, and provision of minimum standards of practice and guidance in clinical practice in countries that did not have such structures in place.

## Discussion

Overall, the response rate (80%) was very good, exceeding that of the previous survey (66%), with a greater response from low‐wealth countries, especially Africa and the Middle East.

### Critical Care Issues

As with our previous surveys, working conditions (ranked first) and staffing levels (ranked third) remain important issues across the world, and these have been responded to by the WFCCN in the recently released revision of the WFCCN workforce guidelines (Bloomer et al., 2019). A literature review of critical care nursing in Canada, Australia, the United Kingdom, New Zealand, and the United States similarly identified critical care nursing staffing, education, and practice standards as priority areas of focus (Gill, Leslie, Grech, & Latour, 2012). Teamwork and wages (ranked second and fifth, respectively) remain important issues globally. There are many discussions in the literature regarding conflict and tension between nursing and medicine, and there is no doubt this does occur in critical care (Kvande, Lykkeslet, & Storli, 2017; Rose, 2011); however, our experience over the years has demonstrated that good teamwork and respectful cooperation between the professions is strong in many critical care units and remains a priority for nursing.

Our worldwide surveys continue to provide evidence of some key differences in priority between wealthy and less wealthy countries. These findings help the WFCCN and other regional federations to more precisely tailor their activities relevant to the local CCN population. We contend that these differences should engender policy variations that are sensitive to our findings in low, middle, and higher wealth regions. For example, the need for formal practice standards and guidelines was the fourth most important issue for critical care nurses globally, but it was the most important issue for low‐ and middle‐wealth countries and was ranked sixth for high‐wealth countries (see Table 1). With the increasing accessibility to online materials and advanced educational tools, this priority is diminishing in importance in developed healthcare systems but remains a top priority in the rest of the world, since low‐ and middle‐wealth settings are still challenged to access standards and guidelines (Murthy & Adhikari, 2013). A similar result was found concerning the need for formal credentialing processes. High‐income countries ranked this the least important, whereas middle‐ and low‐wealth countries ranked it fifth and third, respectively. Anecdotal information via the WFCCN as well as comments from low‐ and middle‐income representatives indicate a view that credentialing will lead to greater professional recognition and thereby increased remuneration. In addition, it is indicative of the fact that many higher income countries already have well‐established professional credentialing processes, whereas others do not. Likewise, standards for education courses was ranked second in low‐wealth countries, and tenth in middle‐ and high‐wealth countries. This is a perennial concern, since poor education is associated with poor patient outcomes (Murthy & Adhikari, 2013). We anticipate that as the Internet and online educational materials become more accessible to nurses in the developing world, these challenges will gradually be overcome. In this regard, one of the most significant current changes is the relatively inexpensive and fast movement of large amounts of data, including educational material and other helpful information to all parts of the world, including those regions previously denied access (Williams et al., 2015). Furthermore, there is little additional cost involved with educational standards and materials developed in predominantly wealthy western countries being made freely available to CCN colleagues in regions that have hitherto been denied access due to resource limitations. In this context, global sharing of resources is very relevant to the mission of the WFCCN and other policy leaders. Amongst other resources, the WFCCN journal Connect is available free of charge and the WFCCN has recently published an online open‐access text book (Goldsworthy, Kleinpell, & Williams, 2019) covering many of the important clinical and professional topics identified as priorities in past surveys. Global sharing of CCN resources is to be encouraged so that knowledge equity can become a reality in developing countries.

### Critical Care Nursing Organization Issues

Critical care nursing organizations are required to provide professional representation and advocacy. This is a very broad responsibility and requires each CCNO to be up to date with the specific needs and aspirations of its professional group. This survey provides global perspectives and themes that may be useful to CCNOs; however, it is highly recommended that national CCNOs conduct their own survey of members and colleagues to provide a contemporary evidence base and mandate for the priorities they choose to focus upon.

The provision of appropriate educational development opportunities for clinical nurses that are relevant to the needs of the clinical service have been identified as most valuable for advancing critical care nursing standards of practice (Deacon et al., 2017).

In low‐wealth countries, the results of our survey indicate that CCNOs need to focus on the provision of practice guidelines and standards for educational courses (which may be sourced from the WFCCN and other international bodies, if made freely available). In addition, local conferences, workshops, education forums, and training and skills acquisition programs are highly valued priorities for low‐wealth countries.

Practice standards and guidelines was rated the most important priority in middle‐wealth countries, while national conferences and workshops or educational forums were most important in wealthier countries. As stated above, the materials and support systems already in place and being effectively used by CCNOs and CCNs in wealthy countries can be easily transferred to low‐ and middle‐wealth countries.

### Policy Expectations of the WFCCN and Other Global Leaders

Our results show that the expectations of the WFCCN are very clear: provision of standards of clinical and professional practice, international conferences, and professional representation are consistent themes. For example, former WFCCN Workforce and Education standards published in 2005 (Williams et al, 2006) were used to support national standards in the United Kingdom (Core Standards Working Party of the Joint Professional Standards Committee, 2013). The provision of a website has been a very important priority in previous surveys but dropped from first to eighth in this most recent study, possibly emphasizing the need for WFCCN to examine the current WFCCN website content and the expressed needs of the CCN community. The respondents to this survey suggested relatively low benefit from the WFCCN investing in the provision of a journal, newsletter, research support, and individualized membership. Awareness of the needs of the profession becomes crucial for global leaders so they are better able to understand and advocate for CCNs and their patients more effectively. From a policy perspective, there is a need for global leaders and their respective organizations to share their existing resources more liberally so that lower‐wealth countries and nurses can benefit from access to their resources, especially educational and practice guideline documents. In addition, the WFCCN must continue to work with other global partners such the International Council of Nurses, World Health Organization, and other international federations of critical care (e.g., medicine and pediatrics) to ensure that shared policy statements are aligned and effective in giving a considered and consistent message and direction to the professions we serve. In providing needed resources and guidance to CCNOs and CCNs such as practice guidelines and access to educational materials, CCNOs and CCNs can help to optimize the level of nursing care provided to patients, and potentially improve outcomes for critically ill patients globally.

## Limitations

Despite our best efforts to find those with experience and standing in leadership roles in their respective countries, of the 82 respondents, only 56 were from countries with a CCNO. Individuals representing the CCNs in their country may not always be well informed of the priorities of the CCNO or CCNs in their country. Although the survey was available in English and Spanish, many respondents not strong in these two languages may not have fully understood all questions. Our best efforts were employed to collectively help those individuals to find a translator; this remains an ongoing challenge that has and will improve over time with better planning, resources, or technology. Nevertheless, potential respondents speaking other languages, those unfamiliar with Survey Monkey, and leaders outside of our network could have been missed.

## Conclusions

Critical care nursing is one of several nursing specialties with an international representative body. This professional infrastructure is starting to show significant benefits as the initiatives of the past two decades are enhanced and improved by successive leadership teams. Since the publication of the previous WFCCN world survey, we have seen the emergence of three new regional CCN federations in Africa, South Asia, and South‐East Asia, with the promise of a Middle‐East federation in the next few years. The challenge of meeting the sometimes‐divergent needs of high‐wealth and middle‐ or low‐wealth countries is beginning to be better understood, acknowledged, and accommodated in policy decisions and priorities of the profession. The findings of this world survey can be used to inform WFCCN policy and strategic plans and influence future priorities and activities of educators, researchers, and nursing and healthcare leaders in order to promote excellence in critical care nursing policy and practice at a global level.

## Supporting information


**Table S1.** Responses by Region and Wealth Group (*n* = 83).
**Table S2.** Categories and Themes of Services and Activities Described in Verbatim Responses.
**Table S3.** Categories and Themes of Position Statements Described in Verbatim Responses.
**Table S4.** Provision and Importance of WFCCN Services and Activities by Wealth Group (*n* = 73).

## References

[jnu12599-bib-0001] Blackwood, B. , Albarran, J. , & Latour, J. (2011). Research priorities of adult intensive care nurses in 20 European countries: A Delphi study. Journal of Advanced Nursing, 67(3), 550–562. 21091912 10.1111/j.1365-2648.2010.05512.x

[jnu12599-bib-0002] Bloomer, M. J. , Fulbrook, P. , Goldsworthy, S. , Livesay, S. L. , Mitchell, M. L. , Williams, G. , & Friganovic, A. (2019). World Federation of Critical Care Nurses 2019 position statement: Provision of a critical care nursing workforce. Connect: The World of Critical Care Nursing, 13(1), 3–7. 10.1891/1748-6254.13.1.3

[jnu12599-bib-0003] Central Intelligence Agency . (2018). The world factbook. Country comparison: GDP per capita (PPP). Retrieved from https://www.cia.gov/library/publications/resources/the‐world‐factbook/rankorder/2004rank.html

[jnu12599-bib-0004] Core Standards Working Party of the Joint Professional Standards Committee . (2013). Core standards for intensive care units. London, UK: The Faculty of Intensive Care Medicine/The Intensive Care Society UK Retrieved from https://www.ficm.ac.uk/sites/default/files/Core%20Standards%20for%20ICUs%20Ed.1%20(2013).pdf

[jnu12599-bib-0005] Deacon, K. S. , Baldwin, A. , Donnelly, K. A. , Freeman, P. , Himsworth, A. P. , Kinoulty, S. M. , … Witton, N. (2017). The national competency framework for registered nurses in adult critical care: An overview. Journal of Intensive Care Society, 18(2), 149–156. 10.1177/1751143717691985 PMC5606424 28979563

[jnu12599-bib-0006] Gill, J. F. , Leslie, G. D. , Grech, C. , & Latour, J. M. (2012). A review of critical care nursing staffing, education and practice standards. Australian Critical Care, 25(4), 224–237. 10.1016/j.aucc.2011.12.056 22306291

[jnu12599-bib-0007] Goldsworthy, S. , Kleinpell, R. , & Williams, G. (Eds.). (2019). International best practices in critical care nursing (2nd ed.). Dayboro, Australia: World Federation of Critical Care Nurses Retrieved from http://wfccn.org/ebook/

[jnu12599-bib-0008] Jamison, D. T. , Gelband, H. , Horton, S. , Jha, P. , Laxminarayan, R. , Mock, C. N. , & Nugent, R. (Eds). (2018). Disease control priorities: Improving health and reducing poverty. Disease control priorities (3rd ed., Vol. 9). Washington, DC: World Bank Retrieved from http://documents.worldbank.org/curated/en/527531512569346552/pdf/121615‐PUB‐HOLD‐embargo‐date‐is‐12‐6‐2017‐ADD‐BOX‐405304B.pdf 30212058

[jnu12599-bib-0009] Kvande, M. , Lykkeslet, E. , & Storli, S. L. (2017). ICU nurses and physicians dialogue regarding patients clinical status and care options—A focus group study. International Journal of Qualitative Studies on Health and Well‐Being, 12(1), 1267346 10.1080/17482631.2016.1267346 28452605 PMC5328360

[jnu12599-bib-0010] Murthy, S. , & Adhikari, N. K. (2013). Global health care of the critically ill in low‐resource settings. Annals of the American Thoracic Society, 10(5), 509–513. 10.1513/AnnalsATS.201307-246OT 24161054

[jnu12599-bib-0011] Murthy, S. , Leligdowicz, A. , & Adhikari, N. K. J. (2015). Intensive care unit capacity in low‐income countries: A systematic review. PLoS ONE, 10(1), e0116949 10.1371/journal.pone.0116949 25617837 PMC4305307

[jnu12599-bib-0012] NHS Wales . (2013). Delivery framework: 2013–14 and future plans. Crown Copyright. Cardiff: Welsh Government Retrieved from http://www.wales.nhs.uk/sitesplus/documents/1064/Delivery%20Framework%202013%2D14.pdf

[jnu12599-bib-0013] Rose, L. (2011). Interprofessional collaboration in ICU. How to define? Nursing in Critical Care, 16(1), 5–10. 10.1111/j.1478-5153.2010.00398.x 21199549

[jnu12599-bib-0014] Schell, C. O. , Gerdin Wärnberg, M. , Hvarfner, A. , Höög, A. , Baker, U. , Castegren, M. , & Baker, T. (2018). The global need for essential emergency and critical care. Critical Care, 22(1), 284–284. 10.1186/s13054-018-2219-2 30373648 PMC6206626

[jnu12599-bib-0015] Stelfox, H. T. , Niven, D. J. , Clement, F. M. , Bagshaw, S. M. , Cook, D. J. , McKenzie, E. , … Critical Care Strategic Clinical Network, Alberta Health Services . (2015). Stakeholder engagement to identify priorities for improving the quality and value of critical care. PLoS ONE, 10(10), e0140141 10.1371/journal.pone.0140141 26492196 PMC4619641

[jnu12599-bib-0016] Williams, G. , Chaboyer, W. , Thorsteinsdottir, R. , Fulbrook, P. , Shelton, C. , Chan, D. , & Wojner, A. (2001). Worldwide overview of critical care nursing organizations and their activities. International Nursing Review, 48(4), 208–217. 11775754 10.1046/j.1466-7657.2001.00100.x

[jnu12599-bib-0017] Williams, G. , Fulbrook, P. , Kleinpell, R. , Schmollgruber, S. , & Alberto, L. (2015). Critical care nursing organizations and activities: A fourth worldwide review. International Nursing Review, 62(4), 453–461. 26303926 10.1111/inr.12205

[jnu12599-bib-0020] Williams, G. , Schmollgruber, R.N. , & Alberto, L. (2006). Consensus Forum: Worldwide Guidelines on the Critical Care Nursing Workforce and Education Standards. Critical Care Clinics, 22, 393–406. 16893727 10.1016/j.ccc.2006.03.010

[jnu12599-bib-0018] World Federation of Critical Care Nurses . (2005a). Declaration of Buenos Aires: Position statement on the provision of critical care nursing workforce. Connect: The World of Critical Care Nursing, 4(2), 28–29. Retrieved from https://connect.springerpub.com/content/sgrwfccn/3/4/94.full.pdf

[jnu12599-bib-0019] World Federation of Critical Care Nurses . (2005b). Declaration of Madrid: Position statement on the preparation of critical care nurses (version 2). Connect: The World of Critical Care Nursing, 4(2), 30–31. Retrieved from https://connect.springerpub.com/content/sgrwfccn/3/4/92.full.pdf

