# Supplementary material for: The Fifth International Survey of Critical Care Nursing Organizations: Implications for Policy
Source: J Nurs Scholarsh. 2020 Oct 22;52(6):652–60. doi: 10.1111/jnu.12599 (PMC7756856; doi:10.1111/jnu.12599)
Supplement: Supplementary file 1 — Table S1. Responses by Region and Wealth Group (n = 83). Table S2. Categories and Themes of Services and Activities Described in Verbatim Responses. Table S3. Categories and Themes of Position Statements Described in Verbatim Responses. Table S4. Provision and Importance of WFCCN Services and Activities by Wealth Group (n = 73). [file JNU-52-652-s001.docx]

Table S4: Provision and Importance of WFCCN Services and Activities by Wealth Group (*n* = 73)

| **Importance of activity/service** | | | | | | | | |
| --- | --- | --- | --- | --- | --- | --- | --- | --- |
| **Description** | **Rank 2013**  **(*n* = 55)** | **Rank 2017**  **(*n* = 75)** | **Overall score (*n* = 75)** | | | **Mean score (*SD*) by wealth group** | | |
|  |  |  | **Mean (*SD*)** | **95% CI** | **Range** | **Top third**  **(*n* = 27)** | **Middle third**  **(*n* = 29)** | **Lower Third**  **(*n* = 17)** |
| **Standards for clinical practice** | 3 | ↑ 1 | 8.99 (2.17) | 8.48-9.49 | 1-10 | 8.59 (2.24) | 9.55 (1.70) | 8.65 (2.64) |
| **International conferences** | 4 | ↑ 2 | 8.88 (1.83) | 8.45-9.30 | 1-10 | 8.67 (1.57) | 8.90 (2.30) | 9.18 (1.24) |
| **Standards for professional practice** | 2 | ↓ 3 | 8.86 (2.18) | 8.35-9.37 | 1-10 | 8.52 (2.31) | 9.34 (1.82) | 8.59 (2.50) |
| **Professional representation** | 6 | ↑ 4 | 8.64 (1.95) | 8.19-9.10 | 1-10 | 7.93 (2.56) | 9.10 (1.32) | 9.00 (1.46) |
| **Study/education grants** | 8 | ↑ 5 | 8.58 (1.96) | 8.12-9.03 | 2-10 | 8.22 (2.21) | 8.72 (1.87) | 8.88 (1.69) |
| **Journal** | 5 | ↓ 6 | 8.26 (2.10) | 7.77-8.75 | 1-10 | 7.67 (2.11) | 8.66 (2.18) | 8.53 (1.77) |
| **Research grants** | 9 | ↑ 7 | 8.26 (2.21) | 7.74-8.78 | 2-10 | 7.78 (2.26) | 8.79 (1.61) | 8.12 (2.87) |
| **Website** | 1 | ↓ 8 | 8.41 (2.14) | 7.91-8.91 | 1-10 | 7.78 (2.31) | 8.93 (1.56) | 8.53 (2.55) |
| **Initiate, conduct or lead research studies** | 7 | ↓ 9 | 7.99 (2.53) | 7.40-8.58 | 1-10 | 7.56 (2.79) | 8.34 (2.42) | 8.06 (2.28) |
| **Newsletter** | 10 | = 10 | 7.71 (2.45) | 7.14-8.28 | 1-10 | 7.04 (2.56) | 8.28 (2.12) | 7.82 (2.68) |
| **Individual membership of WFCCN** | 11 | = 11 | 6.99 (2.21) | 6.29-7.68 | 1-10 | 5.48 (3.25)* | 7.79 (2.38) | 8.00 (2.60) |

**p* = 0.009. CI, confidence interval; *SD* standard deviation.

Table S1. Responses by Region and Wealth Group (*n* = 83)

| **Wealth group** | **Mean (range) GDP per capita (PPP) USD** | **Region** | | | | |
| --- | --- | --- | --- | --- | --- | --- |
|  |  | **Africa**  *n* = 17 | **Americas**  *n* = 18 | **Asia Pacific**  *n* = 14 | **Europe**  *n* = 20 | **Middle East**  *n* = 13 |
| **High**  (*n* = 31) | $50,532  ($27500-$124900) |  | Canada, Puerto Rico, United States | Australia, Hong Kong, Japan, New Zealand, Singapore | Austria, Cyprus, Denmark, Finland, France, Germany, Greenland, Hungary, Iceland, Norway, Poland, Slovenia, Spain, Sweden, Switzerland, United Kingdom | Bahrain, Israel, Kuwait, Oman, Qatar, Saudi Arabia, United Arab Emirates |
| **Middle**  (*n* = 31) | $14,610  ($7700-$24900) | Botswana, Libya, Namibia, South Africa | Argentina, Brazil, Chile, Colombia, Costa Rica, Cuba, Ecuador, Guatemala, Mexico, Paraguay, Peru, Saint Lucia, Uruguay, Venezuela | China, Fiji, Philippines, Sri Lanka, Turkey | Bosnia and Herzegovina, Croatia, Macedonia, Serbia | Egypt, Iran, Jordan, Lebanon |
| **Low**  (*n* = 20) | $3,525  ($900-7200) | Cameroon, Congo, Ghana, Kenya, Lesotho, Liberia, Malawi, Nigeria, Sierra Leone, South Sudan, Uganda, Zambia, Zimbabwe | Bolivia | Cambodia, India, Nepal, Pakistan |  | Afghanistan, Palestine |

Table S2. Categories and Themes of Services and Activities Described in Verbatim Responses

| **Categories** | **Themes** |
| --- | --- |
| Establishment of a professional organisation | Advocacy, collaboration and representation |
| Professional recognition |  |
| Professional representation |  |
| WFCCN membership |  |
| Communication | Communication and networking |
| Communication to membership |  |
| International exchange |  |
| Networking |  |
| Conduct research | Research support |
| Research |  |
| Research grants |  |
| Research support |  |
| Clinical guidelines | Supporting critical care education and practice |
| Conference/travel grants |  |
| Conferences |  |
| Education and training |  |
| Education grants |  |
| Education standards |  |
| Education standards/competencies |  |
| International conferences |  |
| Scholarships |  |
| Conference/travel grants |  |
| Workforce standards |  |
| Support critical care education |  |
| Connect Journal to be indexed in pub med | Supporting publication |

Table S3. Categories and Themes of Position Statements Described in Verbatim Responses

| **Categories** | **Themes** |
| --- | --- |
| Advanced practice | Advance practice |
| Admission to ICU | Clinical guidelines |
| Anaesthesia |  |
| Bundles of care |  |
| Cardiac care |  |
| Clinical guidelines |  |
| Early mobility |  |
| Emergency nursing |  |
| End of life care |  |
| Essential care |  |
| Extracorporeal life support |  |
| Family support |  |
| Infection control |  |
| Monitoring |  |
| Patient safety |  |
| Preoperative care |  |
| Quality improvement |  |
| Renal care |  |
| Research translation |  |
| Respiratory support |  |
| Sepsis care |  |
| Vascular care |  |
| Competence standards | Competencies, education and practice standards |
| Education standards |  |
| Practice standards |  |
| Scope of practice |  |
| Specialty education |  |
|  |  |
| Regulation | Regulation |
| Action research | Research |
| Burnout | Workforce guidelines |
| Recruitment and retention |  |
| Staffing |  |
| Working conditions |  |
| Miscellaneous | Miscellaneous |
